# Commentary: Depletion of the Fragile X Mental Retardation Protein in Embryonic Stem Cells Alters the Kinetics of Neurogenesis

**DOI:** 10.3389/fnmol.2017.00029

**Published:** 2017-02-07

**Authors:** Cara J. Westmark

**Affiliations:** Department of Neurology, University of WisconsinMadison, WI, USA

**Keywords:** APP, FMRP, fragile X syndrome, secretases, neurogenesis

Fragile X syndrome (FXS) is a neurodevelopmental disorder characterized by cognitive impairment, attention deficit, hyperactivity, anxiety, unstable mood, autistic behaviors, language delay, and seizures (Hagerman et al., 2010). This X-linked chromosome disorder is the most common known cause of autism with 30% of boys meeting full autism criteria (Harris et al., 2008). In the majority of cases, FXS is caused by a trinucleotide repeat expansion (CGG) in the *FMR1* gene, which causes loss of expression of fragile X mental retardation protein (FMRP) (Santoro et al., 2011). FMRP is an RNA binding protein that plays a critical role in synaptic protein synthesis. There are medications for managing symptoms of FXS, but there are no disease specific therapies and no cure.

The main challenges confronting the FXS field include early diagnosis, validating outcome measures for clinical trials, and identifying viable treatment targets. In terms of early diagnosis, population wide screening is feasible. Recent advances in *FMR1* allele analysis allow rapid and inexpensive assessment of CGG repeat size, the number of AGG interruptions, and methylation status from blood or saliva samples (Hayward et al., 2016). However, a FXS genetic test is not included in the newborn screening (NBS) panel in the United States due to ethical debates regarding screening for genetic disorders where no therapeutic intervention exists and contemporaneous identification of premutation carriers. Proponents of FXS NBS argue that screening is needed for early detection and intervention (Tassone, 2014). Numerous disease mechanism-based drugs are in clinical trials for FXS and early intervention may be required for better therapeutic efficacy. Preliminary results indicate that babies with *FMR1* premutations exhibit an altered developmental trajectory on measures of nonverbal communication and hyperresponsivity to sensory experiences (Wheeler et al., 2016). Thus, early diagnosis could benefit both full and premutation carriers.

With regard to the urgent need to validate outcome measures for FXS clinical trials, recent trials failed on primary endpoints (Berry-Kravis et al., 2013, 2016). Soluble amyloid precursor protein alpha (sAPPα) is elevated in plasma of autistic children and can be detected in human umbilical cord blood supporting feasibility of this APP metabolite as an early diagnostic autism biomarker (Sokol et al., 2006; Bailey et al., 2008). APP metabolites are also altered in FXS (Westmark et al., 2016), and a recent trial with acamprosate indicates that sAPPα is responsive to drug treatment (Erickson et al., 2014).

Concerning identification of viable treatment targets, excessive signaling through metabotropic glutamate receptor 5 (mGluR_5_) leads to increased translation of numerous synaptic proteins and altered plasticity in FXS (Bear et al., 2004). Many of these proteins are under investigation as potential FXS drug targets. Of relevance herein, two overexpressed proteins are APP and amyloid-beta (Westmark and Malter, 2007), which have been well-studied in Alzheimer's disease (AD). Accumulating evidence suggests that dysregulated levels of APP metabolites contribute to FXS pathology (Figure 1), supporting the hypothesis that pharmaceuticals under study for modulation of APP and amyloid-beta in AD may be viable therapeutic strategies for FXS (Westmark et al., 2013; Pasciuto et al., 2015).

**Figure 1:**
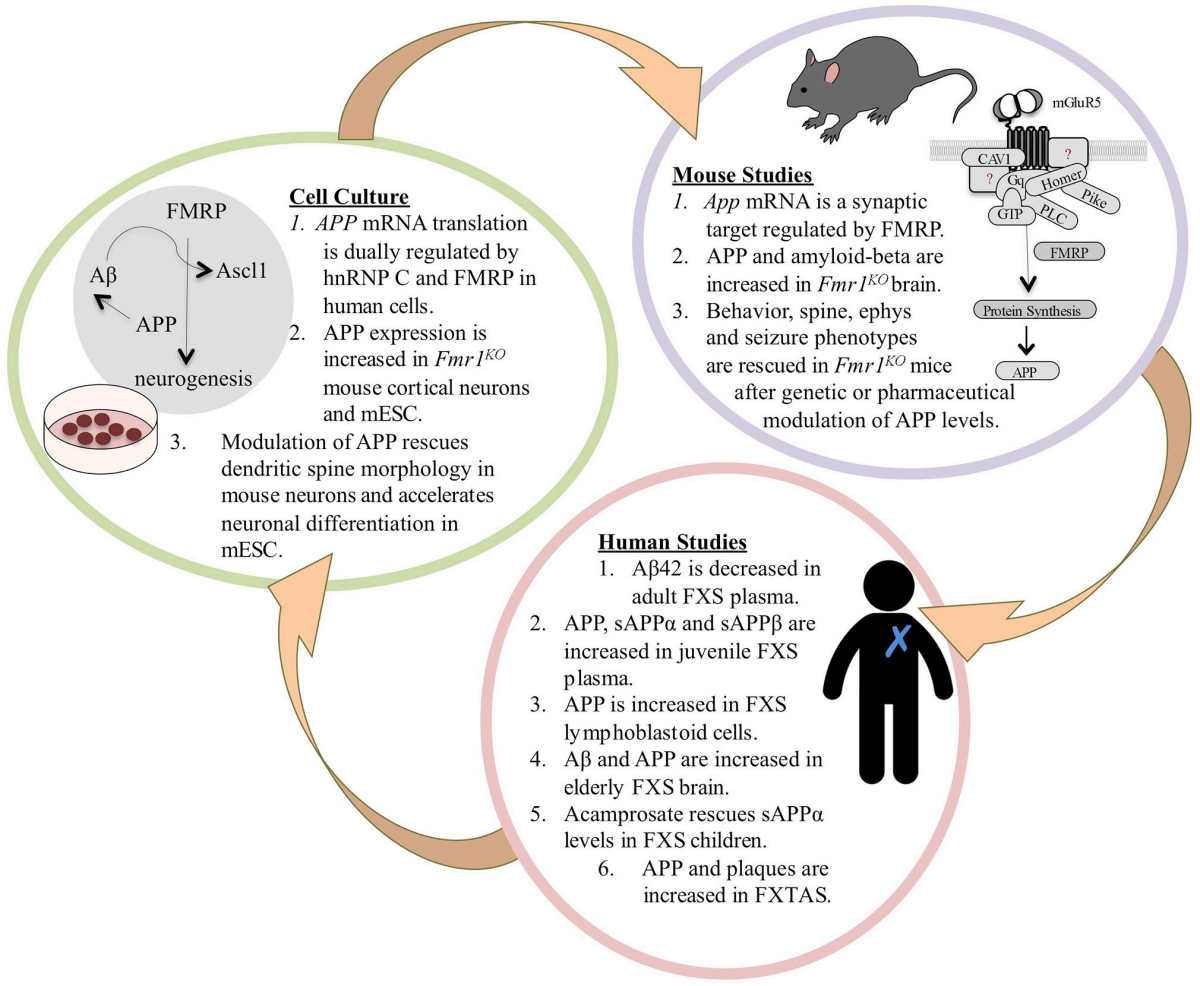
**The APP Theory of FXS**. FXS is a debilitating genetic disorder with no cure and few therapeutic options. Excessive signaling through mGluR_5_ leads to the increased translation of numerous synaptic proteins and exaggerated long-term depression (LTD) in *Fmr1*^*KO*^ mice (Huber et al., 2002; Bear et al., 2004). Two of the overexpressed proteins are APP and its metabolite amyloid-beta (Westmark and Malter, 2007), which have been well-studied in Alzheimer's disease (AD). Accumulating evidence suggests that dysregulated levels of APP and its catabolites contribute to FXS pathology. Multiple recent FXS clinical trials have failed on their primary endpoints indicating that there is a compelling need for validated biomarkers and outcome measures in the field. We hypothesize that APP and its metabolites may be viable blood-based biomarkers that are responsive to drug treatment in FXS, and that pharmaceuticals under study for the modulation of APP and amyloid-beta in AD may be viable therapeutic candidates for FXS. In mice, FMRP binds to a guanine-rich region in the coding region of *App* mRNA and regulates protein translation through mGluR_5_ signaling (Westmark and Malter, 2007). APP and amyloid-beta levels are elevated in *Fmr1*^*KO*^ brain (Westmark and Malter, 2007; Liao et al., 2008; Pasciuto et al., 2015); and behavior, dendritic spine, electrophysiology, and seizure phenotypes are rescued after genetic or pharmaceutical modulation of APP levels in *Fmr1*^*KO*^ mice (Westmark et al., 2011; Pasciuto et al., 2015). These data prompted studies in human samples to determine if APP metabolites may be viable biomarkers for drug efficacy in FXS. In humans, there are altered levels of APP metabolites in FXS blood plasma, lymphoblastoid cells, and brain (Westmark et al., 2011; Pasciuto et al., 2015; Ray et al., 2016); and sAPPα levels are responsive to drug treatment in FXS children (Erickson et al., 2014). FXS is a family of disorders where older premutation carriers can develop fragile X tremor-ataxia syndrome (FXTAS). Elderly FXTAS subjects have elevated *APP* mRNA in blood and APP and amyloid plaques in brain; aged FXTAS knockin mice exhibit elevated brain APP (Tassone et al., 2012; Mateu-Huertas et al., 2014; Renoux et al., 2014). Findings from the mouse and human studies prompted cell culture experiments. In human neuroblastoma cells, there is dual regulation of APP mRNA translation by the RNA binding proteins hnRNP C and FMRP, which compete for binding the guanine-rich regulatory element in the coding region of the message (Lee et al., 2010). There is increased expression of APP in *Fmr1*^*KO*^ mouse cortical neurons and mESC; and genetic, lentiviral or pharmaceutical modulation of APP rescues spine morphology and accelerated neurogenesis (Westmark and Malter, 2007; Westmark et al., 2011; Pasciuto et al., 2015; Khalfallah et al., 2017). FMRP depletion in mESC leads to increased expression of APP and *Ascl1*, which leads to accelerated neuronal differentiation (Khalfallah et al., 2017). Ascl1 is a transcription factor, and of interest, amyloid-beta is a putative transcription factor for *APP* and *BACE1* (Maloney and Lahiri, 2011) and upregulates Ascl1 expression (Uchida et al., 2007). Thus, enhanced transcriptional and translational events mediated by Ascl1, APP and amyloid-beta in the absence of FMRP could drive accelerated neurogenesis in FXS. The FXS mESC model developed by the Bardoni laboratory could be utilized to study cell signaling events at the earliest stage of FXS pathology, including APP synthesis and processing, and be developed into a high throughput assay for drug testing including secretase modulators. Bench-to-bedside plans would need to include validation of identified targets and drugs in future animal and human studies. Overall, this novel mESC model offers a timely tool to study the early events of FXS pathogenesis including the expression and processing of APP.

In their *Stem Cell*s article, Khalfallah and colleagues report the development of a mouse embryonic stem cell (mESC)-based FXS disease model. Specifically, they generated an isogenic stable cell line by targeted knockdown of the *Fmr1* gene with a short hairpin (sh)RNA, and used this model to demonstrate that decreased expression of FMRP triggers accelerated differentiation of neurons and elevated expression of the transcription factor Ascl1/Mash1 and the membrane glycoprotein APP (Khalfallah et al., 2017). *Fmr1* mRNA and protein levels were specifically and significantly reduced in *shFmr1* mESC compared to control cells while expression of FMRP homologs was unaffected. *Fmr1* knockdown did not alter mESC morphology or proliferation; however, there was premature generation of neural progenitors as evidenced by a rosette-like morphology at 4 days *in vitro* and altered expression of neuro-specific markers. The authors confirmed that neurogenesis was accelerated *in vivo* in *Fmr1*^*KO*^ mouse embryonic brain. They further showed that accelerated neurogenesis in the *shFmr1* mESC model was rescued by genetic introduction of the human *FMR1* gene or pharmacological treatment with BACE-1 inhibitor LY2811376.

Neurogenesis is the process through which neurons are generated from neural stem and progenitor cells. Both FMRP a*n*d APP have evolutionarily conserved roles in regulating embryonic and adult neurogenesis (Hayashi et al., 1994; Ohsawa et al., 1999; Caille et al., 2004; Callan et al., 2010; Luo et al., 2010; Demars et al., 2011; Nicolas and Hassan, 2014; Wang et al., 2014, 2016; Faulkner et al., 2015; Halevy et al., 2015). FMRP regulates the translation of both Ascl1 (Fahling et al., 2009) and APP (Westmark and Malter, 2007; Lee et al., 2010). Ascl1 is involved in the transcriptional regulation of genes associated with all major steps of neurogenesis (Castro et al., 2011). APP expression, trafficking, and processing are dynamically regulated during neuronal differentiation (Bergstrom et al., 2016; Ramaker et al., 2016). The APP metabolite amyloid-beta upregulates expression of Ascl1 (Uchida et al., 2007) and drives the differentiation of progenitor cells toward a neuronal lineage (Calafiore et al., 2006). Thus, defective crosstalk among Asc1 and APP metabolites in the absence of FMRP likely contributes to accelerated neurogenesis in FXS. Early interventions targeted at normalizing this signaling pathway could promote normal brain development.

The *shFmr1* mESC model developed by the Bardoni laboratory overcomes the inherent ethical and scientific problems associated with human *FMR1* embryonic stem cells (hESC), most of which carry the methylated and silenced full mutation and/or are mosaic in CGG-repeat length and exhibit residual FMRP expression. In essence, this elegant work addresses the three main challenges of the FXS field by: (1) developing a *Fmr1* knockdown stem cell model that allows study of the earliest events of neurogenesis to support NBS and early intervention; (2) identifying a role for APP in the kinetics of neurogenesis, which supports the development of APP metabolites as potential FXS biomarkers; and (3) demonstrating rescue of *shFmr1* mESC morphology with a BACE-1 inhibitor thus promoting study of APP and secretases as therapeutic targets for FXS.

Khalfallah and colleagues contribute a vital piece to the FXS puzzle in describing development of a mESC model that allows study of early molecular events underlying disease development and provides a new platform for preclinical drug testing. Substantial data is provided validating the morphological and molecular characteristics of the *shFmr1* mESC as well as demonstrating rescue of phenotypes by re-introduction of FMRP or by targeting APP processing via inhibition of BACE-1. Future experiments could examine expression of various APP metabolites on neurogenesis, compare BACE-1 and mGluR_5_ inhibitors, confirm FXS signaling pathways in this early disease-stage model, and transfect plasmids carrying varying length CGG repeats in the human *FMR1* gene to mimic the repeat expansion aspect of the disorder.

## Author contributions

The author confirms being the sole contributor of this work and approved it for publication.

### Conflict of interest statement

The author declares that the research was conducted in the absence of any commercial or financial relationships that could be construed as a potential conflict of interest.
